# Could Poor Periodontal Status be a Warning Sign for Worse Pregnancy Outcome?

**DOI:** 10.3290/j.ohpd.a43356

**Published:** 2020-02-12

**Authors:** Tibor Novák, Gábor Németh, Zoltan Kozinszky, Edit Urbán, István Gorzó, Márta Radnai

**Affiliations:** a Clinical Doctor, Department of Obstetrics and Gynecology, University of Szeged, Szeged, Hungary. Selection and examination of the patients.; b Professor, Department of Obstetrics and Gynecology, University of Szeged, Szeged, Hungary. Selection and examination of the patients.; c Clinical Doctor, Department of Obstetrics and Gynecology, Blekinge Hospital, 37185 Lasarettsvagen Karlskrona, Sweden. Performed the statistical analysis.; d Clinical Doctor, Institute of Clinical Microbiology, University of Szeged, Szeged, Hungary. Helped with the experimental design.; e Professor, Faculty of Dentistry, University of Szeged, Szeged, Hungary. Starting and sustaining the hypothesis.; f Professor, Faculty of Medicine, Department of Prosthodontics, University of Pécs, Pécs, Hungary. Selection and examination of the patients.

**Keywords:** birth weight, periodontal status, pregnancy, prematurity

## Abstract

**Purpose:**

The aim of the present study was to analyse the role of the main diagnostic signs of poor periodontal status, probing depth (PD) ≥4 mm and bleeding on probing (BOP) ≥50%, both simultaneously and individually in preterm birth (PB) and their effect on the birth weight (BW). Prematurity is a major health concern and it is the leading contributing factor to neonatal morbidity and mortality worldwide. Conflicting results exist on the relation between poor maternal, periodontal status and adverse pregnancy outcome, including preterm deliveries and low birth weight (LBW).

**Materials and Methods:**

Seventy-seven PBs and 165 deliveries at term were analysed out of 242 patients. The perinatal factors such as gestational age (GA) and BW were analysed by BOP, categorised as ≥50% (high BOP) vs <50% (non-high BOP) and PD ≥4 mm vs <4 mm, in combination or separately.

**Results:**

The obtained results suggest that maternal periodontal inflammation, represented particularly by BOP, might be a triggering factor and can be associated with PB and LBW. For women with high BOP the adjusted odds ratio (AOR) for LBW was 2.28-fold and they were likely to have PB, with a 2.02-fold higher rate.

**Conclusion:**

An increasing tendency of BOP seemed to be associated with a tendency to PB and LBW. Further investigations are necessary to underline this relationship, but the role of good oral health status in general, and particularly in case of pregnancy, is unequivocal.

Prematurity is a major health concern and it is the leading contributing factor to neonatal morbidity and mortality worldwide, even in developed countries. Conflicting results exist on the relation between poor oral health status, periodontal disease and adverse pregnancy outcome, including preterm deliveries and low birth weight (LBW). These potential connections have been examined since the 1990s. There are several classification systems regarding periodontal diseases and conditions, including pregnancy gingivitis, which is a commonly recognised disease induced by plaque and modified by systemic factors.^1^ The characteristic signs for this entity are hyperaemia and oedema (enlargement) of the gingiva, which bleeds easily, and hyperplasia of interdental papilla leading to pseudo-pockets with increased probing depth (PD).^15^ The exact mechanisms by which the hormonal changes during pregnancy, especially the modifications of the level of free plasma oestrogens and progesterone, are involved in increasing the severity of gingival symptoms during pregnancy are still not clear. Probably one of the triggering factors are the sexual hormonal changes causing a more susceptible environment for colonisation of gingiva with bacteria (especially Prevotella species), an increased capillary permeability and higher gingival crevicular flow rate, more dilated capillary network, decrease in the keratinisation of the marginal gingival epithelium, leading eventually to an increased bleeding tendency.^15^ However, these inflammatory changes on the gingival site via the haematogenous route can affect the pregnant uterus, inducing symptoms of threatening preterm delivery, causing preterm uterine activity and/or premature rupture of the membrane, which may or may not be associated with cervical dilation.

Some studies have shown a statistically significant association between periodontal status^16^ and preterm birth (PB), whereas others could not verify this.^4,9^

The conflicting results on the relationship between periodontal disease and PB and/or LBW remains controversial because there is no complete consensus concerning the signs of periodontal disease implementing PD, clinical attachment loss, and bleeding on probing (BOP)^9^ in the various studies. Furthermore, the studies have disparities in the socioeconomic and obstetric background of the enrolled maternal population. However, a strong association was also^2^ shown between periodontitis and poor pregnancy outcomes, and on the other hand, it was concluded that severe anaemia and periodontal infection may have an adverse effect on pregnancy and fetal development.^7^

One study^21^ suggested that BOP at >20%, as an indicator of the inflammation of the periodontal tissues, correlates negatively with the gestational length, birth weight (BW) and the rate of LBW. Some other studies observed a statistically significant association between the PB and periodontitis and PD ≥5 mm.^13,14^ The purpose of the present study was to analyse the role of the main diagnostic signs of poor periodontal status (PPS) (defined as PD ≥4 mm at least at one site and BOP ≥50% of the teeth in this study) both simultaneously and individually in preterm delivery and neonatal birth weight in Southeast Hungary.

## Materials and Methods

A cross-sectional study was conducted over a period of 2 years in the Department of Obstetrics and Gynecology, University of Szeged, Hungary, a tertiary-level hospital in southeastern Hungary with around 2500 deliveries annually, serving about 1,300,000 inhabitants. The patients were enrolled in two groups: preterm and term one:

1. those who delivered before the completed 37 weeks of gestation (PB group, N = 77); and
2. those who delivered at term (>37 weeks, term birth (TB) group, N = 165).

The examinations were completed within 3 days post-partum. For each preterm delivery, about two term deliveries of the same day were selected by lottery, in concordance with the enrolment criteria. Nine cases in the TB group were also included because of the overnight timing of the deliveries, which started in the previous day, and finished the next.

Some major perinatal characteristics were presented when the patients were compared according to their gestation at delivery and periodontal status. The gestational age (GA) was determined by sonographic measurement of the embryo in the first trimester. PB was classified as birth <37 weeks, while a delivery was categorised as a very preterm birth (VPB) when it occurred between completed 24 and 32 weeks. BW <2500 grams (g) was defined as LBW and <1500 g as very low birth weight (VLBW).

A dental unit installed at the Department of Obstetrics and Gynecology, based on the author’s previous studies concerning the association between maternal PPS and obstetric outcome, was used for the dental examination.^12^ The dental unit enabled precision for the periodontal examinations, because the position of the patients and the lightening were optimal.^17^

All healthy women without any systemic disease, singleton pregnancy and at least 16 teeth presented for threatening PB, diagnosed by shortened cervix of 25 mm with or without any regular uterine contractions before the completed 37 weeks of gestation, were recruited in the PB group. A series of four doses of corticosteroids (6 mg), given in every 12 hours, were administered for all women and oral/intravenous tocolytic agents were used.

Exclusion criteria were as follows: mothers experiencing PB with chronic disease (n = 182), PB complicated with premature ruptures of the membranes and treated with antibiotics (n = 63); patients who received periodontal treatment during their actual pregnancy 2 months before the study (n = 18); fever/subfertility or clinical sign of chorioamnionitis at the time of recruitment (n = 5); fetal death (n = 23); major fetal defect (n = 12); cervical cerclage in situ (n = 3); and refusal to participate (n = 12). Finally, 242 women were included in the study and were divided into two groups.

Periodontal examinations were performed according to the World Health Organization (WHO) guidelines,^11^ by a dentist with extensive experience in dental and periodontal screening. The dentist was blind to the gestational age at delivery. A disposable periodontal probe with a tip diameter of 0.5 mm was used for measuring PD in mm, from the gingival margin to the most apical point of the sulcus/pocket, at six sites of each tooth (ie, mesiobuccal, mid-buccal, distobuccal, mesiolingual, mid-lingual and distolingual), with the exception of the third molars. BOP was recorded at the sites where PD was measured after 15 s on the Yes/No scale. PPS was defined as a pocket with a PD ≥4 mm found at least at one site, and BOP ≥50% of the teeth. These criteria for the assessment of periodontal health were selected from the authors’ previous studies, when gingival bleeding and bigger PD were the most important signs of periodontal inflammation.^16^ Every recruited woman was instructed on good oral hygiene after periodontal examination.

Personal data and sociodemographic status including the educational level and profession were recorded. Educational levels were defined as follows: primary school (8 years), secondary (3 or 4 years trade school or grammar school) and tertiary (university or college). The occupations were categorised as manual worker, other (eg, shop assistant), intellectual/white collar worker or unemployed persons. Adverse habit (eg, smoking) was also recorded.

Perinatal factors (eg, gestational age, birth weight) were analysed by BOP categorised as ≥50% (high BOP (HBOP)) vs <50% (non-high BOP (NHBOP)) and PD ≥4 mm vs <4 mm. The comparison of those who had PPS with those who had healthy periodontium (PH) was also performed.

Statistical analyses were performed using SPSS for Windows, Version 15 (SPSS, Chicago, IL, USA). Univariate comparisons for categorical variables were assessed by χ^2^ tests and independent student’s t tests for continuous variables. Statistical significance was defined at the two-sided p <0.05 level. Odds ratios (ORs) and Cornfield 95% confidence intervals were calculated for categorical variables, whereas odds ratios for continuous variables were evaluated by univariate logistic regression. In multivariate analyses, ORs were adjusted for confounding factors (age, education, smoking status, occupation, residence) using logistic regression for each of the perinatal parameters according to periodontal status.

The patients were informed about the aim of the study and they took part voluntarily, after signing the written consent form. The Human Investigation Review Board of University of Szeged, Albert Szent-Györgyi Clinical Centre, approved the study. This study is reported in accordance with the Strengthening the Reporting of Observational Studies in Epidemiology (STROBE) statement for improving the quality of observational studies.^20^

## Results

The recruited women were divided into two groups according to the time of delivery and the data are presented in Table 1. Seventy-seven (77) women experienced premature delivery, whereas 165 delivered at term. The PB group was significantly older, and it consisted of highly skilled workers, which is in concordance with the tendency that higher educated women have childbirth at a later age. The two groups were similar in terms of educational status, residence and the rate of primiparous women. Statistically significantly more smokers were recorded in the PB group. All registered perinatal indicators were significantly worse in the PB group.

**Table 1:** Sociodemographic and obstetric data

	PB group (N = 77)		TB group (N = 165)		p value
	n	%	n	%	
Age (y, mean ± SD)	29.3 ± 5.2		27.9 ± 4.9		0.046
**Education**					
Primary	14	18.2	20	12.1	0.163
Secondary	36	46.8	98	59.4	
Tertiary	27	35.0	47	28.5	
**Residence**					
Urban	39	50.6	96	58.2	0.33
Rural	38	49.4	69	41.8	
**Occupation**					
Unemployed	20	26.0	45	27.2	0.009
Manual worker	16	20.8	62	37.6	
Other	16	20.8	32	19.4	
Intellectual	25	32.4	26	15.8	
Smoking	14	18.2	8	4.8	0.001
Primiparity	42	54.5	97	58.8	0.58
GA (w, mean ± SD)	34.1 ± 2.5		38.8 ± 1.2		0.001
VPB (<32 w)	10	13.0	0	0	0.001
BW (g, mean ± SD)	2313.1 ± 637.6		3337.2 ± 487.4		0.001
LBW (<2500 g)	45	58.4	7	4.2	0.001
VLBW (<1500 g)	11	14.3	0	0	0.001

PB, preterm birth; TB, term birth; y, years; SD, standard deviation; GA, gestational age at delivery; w, weeks; VBP, very preterm birth; BW, birth weight; g, grams; LBW, low birth weight; VLBW, very low birth weight.

Characteristics of delivery according to periodontal status are presented in Table 2. The mean birth weight of the newborns of women with PPS group was 2803.0 ± 774.5 g, which was lower compared with the periodontally healthy (PH) group, at 3108.6 ± 673.6 g, the difference being statistically significant (p = 0.002, AOR = 0.99). Mothers with PPS delivered more newborns with LBW (p = 0.001, AOR = 2.58), but not with VLBW (p = 0.33, AOR = 1.58) compared with those who had no periodontal problems. The length of pregnancy also differed statistically significantly between the study groups (36.6 ± 3.0 w in the PPS group and 37.7 ± 2.6 w in the PH group, p = 0.011 and AOR = 0.89). The incidence of deliveries at <37 weeks of gestation was statistically significantly higher in the PPS group (42.9% vs 26.7%) (p = 0.017, AOR: 1.95). The difference between the rate of VPB (7.8% in PPS group vs 2.4% in PH group) was not statistically significant (p = 0.078, AOR 3.26).

**Table 2:** Characteristics of delivery according to periodontal status

	PPS (n = 77)		PH (n = 165)		p value	Unadjusted OR (95% CI)	Adjusted OR (95% CI)
	n	%	N	%			
BW (g, mean ± SD)	2803.0 ± 774.5		3108.6 ± 673.6		0.002	0.99 (0.99–0.99)	0.99 (0.99– 0.99)
LBW (<2500 g)	27	35.1	25	15.2	0.001	3.02 (1.61–5.69)	2.58 (1.29– 5.16)
VLBW (<1500 g)	5	6.5	6	3.6	0.33	1.84 (0.54–6.23)	1.58 (0.43– 5.80)
GA (w, mean ± SD)	36.6 ± 3.0		37.7 ± 2.6		0.011	0.88 (0.80–0.97)	0.89 (0.80– 0.99)
PB (<37 w)	33	42.9	44	26.7	0.017	2.06 (1.17–3.64)	1.95 (1.01–3.74)
VPB (<32 w)	6	7.8	4	2.4	0.078	3.40 (0.93–12.4)	3.26 (0.83– 12.8)

PPS, poor periodontal status; PH, peridontally healthy; BW, birth weight; g, grams; LBW, low birth weight; VLBW, very low birth weight; GA, gestational age at delivery; w, weeks; SD, standard deviation; PB, preterm birth; VPB, very preterm birth; OR, odds ratio.

In Table 3, relations between HBOP and selected perinatal parameters are presented. HBOP represent a risk factor for lower BW of the newborn (2830 ± 75 g vs 3118.7 ± 68 g, p = 0.03, AOR: 0.99) at a lower gestational age (36.7 ± 2.9 w vs 37.7 ± 2.6 w, p = 0.014, AOR: 0.88). Regarding LBW, it was 32.2% for the HBOP, and 15.1% for the NHBOP cases (p = 0.003, AOR = 2.28). The differences between the rates of VLBW (p = 0.54) and VPB (p = 0.18) in relation to this periodontal pathology were not notable. PB occurred statistically significantly more often in the HBOP group (42.2%) as compared with the NHBOP group (25.7%) (p = 0.01, AOR = 2.02).

**Table 3:** HBOP (BOP ≥50%) and selected perinatal parameters

	HBOP (N = 90)		NHBOP (N = 152)		p value	Unadjusted OR (95% CI)	Adjusted OR (95% CI)
	N	%	N	%			
BW (g)	2830.0 ± 752.1		3118.7 ± 680.2		0.03	0.99 (0.99–0.99)	0.99 (0.99–0.99)
LBW (<2500 g)	29	32.2	23	15.1	0.003	2.67 (1.43–4.99)	2.28 (1.06–4.89)
VLBW (<1500 g)	5	5.6	6	3.9	0.54	1.43 (0.42–4.83)	1.44 (0.29–7.17)
GA (w ± SD)	36.7 ± 2.9		37.7 ± 2.6		0.014	0.89 (0.81–0.98)	0.88 (0.78–0.99)
PB (<37 w)	38	42.2	39	25.7	0.01	2.12 (1.22–3.69)	2.02 (1.23–4.22)
VPB (<32 w)	6	6.7	4	2.6	0.18	2.64 (0.73–9.63)	4.27 (0.66–27.67)

HBOP, high bleeding on probing >50% of the teeth; NHBOP, non-high BOP <50% of the teeth; BW, birth weight; g, grams; LBW, low birth weight; VLBW, very low birth weight; GA, gestational age at delivery; w, weeks; SD, standard deviation; PB, preterm birth; VPB, very preterm birth; OR, odds ratio.

The association between highest posterior density (HPD) (PD ≥4 mm) and the selected perinatal parameters are presented in Table 4. HPD was a distinguishable factor only for LBW (29.1% for the HPD group vs 15.2% for the NHPD group, p = 0.012, AOR = 0.44) among the perinatal factors.

**Table 4:** Correlation of the HPD (PD ≥4 mm) and selected perinatal parameters

	HPD (N = 110)		NHPD (N = 132)		p value	Unadjusted OR (95% CI)	Adjusted OR (95% CI)
	n	%	N	%			
BW (g)	2932.2 ± 750.1		3077.3 ± 689.7		0.12	1.00 (1.00–1.00)	1.00 (1.00–1.00)
LBW (<2500 g)	32	29.1	20	15.2	0.012	0.44 (0.23–0.82)	0.44 (0.21–0.91)
VLBW (<1500 g)	5.0	4.5	6	4.5	1.00	1.00 (0.30–3.37)	0.79 (0.17–3.67)
GA (w ± SD)	37.1 ± 2.8		37.5 ± 2.7		0.18	1.07 (0.97–1.17)	1.09 (0.97–1.22)
PB (<37 w)	40	36.4	37	28.0	0.17	0.68 (0.40–1.17)	0.57 (0.29–1.13)
VPB (<32 w)	6.0	5.5	4	3.0	0.52	0.54 (0.15–1.97)	0.36 (0.06–2.08)

HPD, high probing depth; NHPD, non-high PD; BW, birth weight; g, grams; LBW, low birth weight; VLBW, very low birth weight; GA, gestational age at delivery; w, weeks; SD, standard deviation; PB, preterm birth; VPB, very preterm birth; OR, odds ratio.

## Discussion

The study results confirmed that PPS, BOP at a cut-off of ≥50% might have an effect on the gestational length and neonatal birth weight, while PD was associated with a subcategory of LBW in threatened PB. By contrast, PD alone did not represent any risk for prematurity.

The obtained results suggested that maternal periodontal inflammation, represented particularly by BOP, might be a triggering factor for threatening PB which can eventually lead to preterm delivery. An increasing tendency of BOP seemed to be associated with a lower GA and BW, which can be explained by two pathophysiological processes affecting the pregnancy outcome and the fetal growth.

1. The direct pathway, when the oral pathogen bacteria and/or their products reach the uterus via hematogenous dissemination from the oral cavity. The hormonal changes during pregnancy cause modification in the microvascular density and permeability of the gingiva. The increased risk of bacteraemia of the Gram-negative microorganisms, which is more pronounced in inflammatory processes in periodontal diseases, is followed by ‘placental seeding’. This pathway was demonstrated in cases where microorganisms exclusively associated with periodontal infection have been cultured from amniotic fluid and neonates.^3,18^
2. The indirect pathway, in which the inflammatory mediators and/or microbial components produced by periodontal tissues circulate to the liver, enhancing cytokine production and acute phase protein responses inducing a systematic inflammatory cascade.^18^ Endotoxins released by microorganisms involved in the periodontal disease are also responsible for triggering cytokine production (primarily locally and also systematically). As a consequence prostaglandin production is stimulated, particularly prostaglandin-E2, an inflammatory factor that might induce parturition both at term or preterm.^3^ The results of the study highlight, in conformity with this theory, that an increased intensity of inflammatory processes, reflected by a HBOP, may exhibit a greater risk of PB and lower BW. Hence, the prevention of the periodontal inflammatory disorders is highly preferable before or during pregnancy in order to avoid the adverse perinatal outcome.^12^ In Hungary dental screening, periodontal assessment and the necessary treatment provided by the family/local dentist are included in the routine prenatal care,^12,17^ however, educating on the importance of the prevention is necessary among young women of childbearing age because many are afraid of dental and periodontal treatment.

A large body of literature strengthens this theory demonstrating similar results on periodontal inflammation,^18^ while many studies failed to find evidence of any connection.^23^ In part the causes of these differences are probably due to the non-uniform methods and definition of periodontal disease and measurements of the periodontal parameters.^9^

Of interest, to the authors’ knowledge no one study has examined the role of BOP solely in the detrimental effect of periodontal diseases on the obstetric outcome. Some results indicated that BOP concomitant with clinical attachment loss^21^ could induce LBW. In line with others^8,10^ this study confirmed also that high BOP combined with high PD (= PPS) was statistically significantly associated with LBW and PB, which was echoed in previous team report too.^17^

On the other hand, the role of urogenital infection as a causing factor for PB is well established, and it may contribute to up to 50% of the cases.^5^ Moreover, the antibiotic treatment of bacterial vaginosis, for example, significantly reduced miscarriage and PB in a randomised controlled trial.^22^ Specifically, in this study, women were recruited with threatened premature delivery with no obvious signs of urogenital infection and/or no antibiotic treatment prior to the periodontal examination, which strengthens the value of this study. Meanwhile, a limitation of the study was that clinical attachment loss was not investigated; its precise measurement needs more time compared with the that of probing depth and the comfort of the women must be considered as well (ie, not making them to sit or lay in the dental chair for a longer time shortly after delivery). Furthermore, to have the risk of bacterial penetration into the gingival tissues, the size of the sulcus/pocket wall is the most important factor and not the actual level of the epithelial attachment.^19^ Also, a disparity in the tendency in smoking and the fact that older age promotes delivery at an earlier gestation with a lower birth weight might also be a subject for a bias.

The limitations of this study may be the relatively small number of the cases and the statistically significant difference between the normal and PB groups. Women in the PB group were little older, however, this difference likely does not have a clinical role. This group included more of those from a highly educated/skilled background, which may be an explanation for the tendency to conceive later, at a more advanced age. The other factor that may have influenced the results was the larger number of smokers in the PB group, a factor which can also be mentioned as a limitation of the study.

## Conclusion

Taken together, the present data have shown that the AOR of LBW is 2.28 times higher for women with HBOP and they are likely to have PB, with a 2.02-fold AOR.

Of course, extended database analysis (including original publications, reporting data from cross-sectional, case–control or prospective cohort epidemiological, non-interventional studies on the field of prematurity and maternal periodontal status) showed that there are modest but statistically significant associations with LBW and PB, and the results are impacted by the periodontitis case definitions.^6^ Further investigations are necessary to underline this relationship, but the role of good oral health in general, and particularly in pregnancy, is unequivocal.

## References

[ref1] Armitage GC (1999). Development of a classification system for periodontal diseases and conditions. Ann Periodontol.

[ref2] Basha S, Shivalinga Swamy H, Noor Mohamed R (2015). Maternal periodontitis as a possible risk factor for preterm birth and low birth weight. A prospective study. Oral Health Prev Dent.

[ref3] Clothier B, Stringer M, Jeffcoat MK (2007). Periodontal disease and pregnancy outcomes: exposure, risk and intervention. Best Pract Res Cl Ob.

[ref4] Fogacci MF, Leão A, Vettore MV (2010). Letter to the editor and the authors’ reply. J Dent Res.

[ref5] Heimonen A, Janket SJ, Kaaja R, Ackerson LK, Muthukrishnan P, Meurman JH (2009). Oral inflammatory burden and preterm birth. J Periodontol.

[ref6] Ide M, Papapanou PN (2013). Epidemiology of association between maternal periodontal disease and adverse pregnancy outcomes – systematic review. J Periodontol.

[ref7] Kothiwale SV, Desai BR, Kothiwale VA, Gandhid M, Konin S (2014). Periodontal disease as a potential risk factor for low birth weight and reduced maternal haemomglobin levels. Oral Health Prev Dent.

[ref8] López NJ1, Da Silva I, Ipinza J, Gutiérrez J (2005). Periodontal therapy reduces the rate of preterm low birth weight in women with pregnancy-associated gingivitis. J Periodontol.

[ref9] Manau C, Echeverria A, Agueda A, Guerrero A, Echeverria JJ (2008). Periodontal disease definition may determine the association between periodontitis and pregnancy outcomes. J Clin Periodontol.

[ref10] Marin C, Segura-Egea JJ, Martınez-Sahuquillo A, Bullon P (2005). Correlation between infant birth weight and mother’s periodontal status. J Clin Periodontol.

[ref11] Mitchell-Lewis D, Engebretson SP, Chen J, Lamster IB, Papapanou PN (2001). Periodontal infections and pre-term birth: early findings from a cohort of young minority women in New York. Eur J Oral Sci.

[ref12] Novák T, Radnai M, Gorzó I, Urbán E, Orvos H, Eller J (2009). Pál. Prevention of preterm delivery with periodontal treatment. Fetal Diagn Ther.

[ref13] Perunovic NDj, Rakic MM, Nikolic LI, Nikolic LI, Jankovic SM, Aleksic ZM (2016). The association between periodontal inflammation and labor triggers (elevated cytokine levels) in preterm birth: a cross-sectional study. J Periodontol.

[ref14] Piscoya M, Ximenez R, Silva G, Jamelli S, Coutinho S (2012). Maternal periodontitis as a risk factor for prematurity. Pediatr Int.

[ref15] Raber-Durlacher JE, van Steenbergen TJM, van der Velden U, de Graaff J, Abraham-Inpinjn L (1994). Experimental gingivitis during pregnancy and post- partum: clinical, endocrinological, and microbiological aspects. J Clin Periodontol.

[ref16] Radnai M, Gorzó I, Urbán E, Eller J, Novák T, Pál A (2006). Possible association between mother’s periodontal status and preterm delivery. J Clin Periodontol.

[ref17] Radnai M, Pál A, Novák T, Urbán E, Eller J, Gorzó I (2009). Benefits of periodontal therapy when preterm birth threatens. J Dent Res.

[ref18] Sanz M, Kornman K, on behalf of working group 4 of the joint EFP/AAP workshop (2013). Periodontitis and adverse pregnancy outcomes: consensus report of the joint EFP/AAP workshop on periodontitis and systemic disease. J Clin Periodontol.

[ref19] Slots J (2003). Update on general health risk of periodontal disease. Int Dent J.

[ref20] STROBE statement. www.strobe-statement.org.

[ref21] Takeuchi N, Ekuni D, Irie K, Furuta M, Tomofuji T, Morita M (2013). Relationship between periodontal inflammation and fetal growth in pregnant women: a cross-sectional study. Arch Gynecol Obstet.

[ref22] Ugwumadu A, Manyonda I, Reid F, Hay P (2003). Effect of early oral clindamycin on late miscarriage and preterm delivery in asymptomatic women with abnormal vaginal flora and bacterial vaginosis: a randomized controlled trial. Lancet.

[ref23] Wang YL, Lion JD, Pan WL (2013). Association between maternal periodontal disease and preterm delivery and low birth weight. Taiwan J Obstet Gyn.

